# Distinct chemokines selectively induce HIV-1 gp120-integrin α4β7 binding via triggering conformer-specific activation of α4β7

**DOI:** 10.1038/s41392-021-00582-8

**Published:** 2021-07-16

**Authors:** Shu Wang, ChangDong Lin, Yue Li, ZhaoYuan Liu, JunLei Wang, YouHua Zhang, ZhanJun Yan, YueBin Zhang, GuoHui Li, JianFeng Chen

**Affiliations:** 1 State Key Laboratory of Cell Biology, Center for Excellence in Molecular Cell Science, Shanghai Institute of Biochemistry and Cell Biology, Chinese Academy of Sciences, University of Chinese Academy of Sciences, Shanghai, 200031 China; 2 Department of Orthopedics, Suzhou Ninth People’s Hospital, Soochow University, Suzhou, 215000 China; 3 State Key Laboratory of Molecular Reaction Dynamics, Dalian Institute of Chemical Physics, Chinese Academy of Sciences, Dalian, 116023 China; 4 School of Life Science, Hangzhou Institute for Advanced Study, University of Chinese Academy of Sciences, Hangzhou, 310024 China

**Keywords:** Infection, Structural biology

**Dear Editor**,

Gut associated lymphoid tissue (GALT) is the principal site where human immunodeficiency virus 1 (HIV-1) replicates. CD4^+^ T cells residing in GALT are predominant targets of HIV-1 during the acute phase of infection. CD4^+^ T cells expressing a high level of gut-homing receptor integrin α4β7 are more susceptible to productive infection by HIV-1.^1^ It has been reported that HIV-1 envelope protein gp120 can bind to integrin α4β7.^2^ Furthermore, the engagement of α4β7 by gp120 on CD4^+^ T cells results in rapid activation of LFA-1, which facilitates efficient cell-to-cell spreading of HIV-1. In macaques, treatment with anti-α4β7 monoclonal antibody efficiently reduces mucosal transmission of simian immunodeficiency virus (SIV), and a combination of antiretroviral drug therapy (ART) with α4β7 antibody treatment effectively prevents the virus rebound after withdrawal of ART.^3^ These findings demonstrate that gp120-α4β7 interaction has an important role in HIV infection.

Integrin α4β7 mediates lymphocyte homing to the gut by binding to mucosal vascular addressin cell adhesion molecule 1 (MAdCAM-1) which expressed on venules in GALT. The critical α4β7 binding motif in MAdCAM-1 is Leu-Asp-Thr (LDT) located in domain 1 (D1), and the core Asp residue is pivotal for integrin binding. Of note, a conserved tripeptide Leu-Asp-Val/Ile (LDV/I) motif in the V2 loop of gp120 is thought to mediate the interaction with α4β7 by mimicking the binding epitopes in MAdCAM-1. In addition to MAdCAM-1, integrin α4β7 can also bind to vascular cell adhesion molecule 1 (VCAM-1). The interaction of integrin with its ligand is dynamically regulated by integrin activation through inside-out signaling, which is associated with a global conformational rearrangement of integrin ectodomains from bent to extended conformation. Integrin extracellular domains exist in at least three distinct global conformational states: bent with a closed headpiece, extended with a closed headpiece, and extended with an open headpiece. The closed and open headpieces have low- and high-affinity for ligands, respectively. The equilibrium among these different states is regulated by integrin inside-out signaling triggered by stimuli such as chemokines. The ligand binding to integrin can also induce outside-in signaling by triggering activation of signal pathways in cells, thus regulate multiple cellular functions.

Our previous study has demonstrated that chemokine CCL25 and CXCL10 activate separate pathways (p38α MAPK/PKCα pathway for CCL25; c-Src/Syk pathway for CXCL10), which stimulates differential phosphorylation states of the integrin β7 tail and distinct talin and kindlin-3 binding patterns, leading to different inside-out activation signals for α4β7.^4^ As a result, CCL25 induces a more extended active conformer of α4β7 that has an increased affinity for MAdCAM-1 but decreased affinity for VCAM-1, however, CXCL10 induces a less extended active conformer with totally opposite affinity changes for both ligands.^5^ These findings indicate that the ligand binding to integrin α4β7 is dependent on the distinct integrin active conformations induced by different chemokines. Although a few studies have provided evidence supporting the binding of gp120 to the active integrin α4β7, whether and how the gp120-α4β7 binding is regulated by physiological stimuli like chemokines remains to be clarified. Moreover, the gp120 binding-induced integrin outside-in signaling calls for an in-depth elucidation.

To investigate the interaction between integrin α4β7 and gp120, we first established CD4 knocked out Jurkat T cell line (CD4^‒^ Jurkat T) to eliminate the binding of gp120 to CD4, and then stably expressed integrin α4β7 in these cells (CD4^‒^α4β7^+^ Jurkat T) (Supplementary Fig. S1a). In the presence of 1 mM Ca^2+^ and 1 mM Mg^2+^, the physiological divalent cations which maintain integrin α4β7 in its inactive state, CD4^‒^α4β7^+^ Jurkat T cells did not adhere to the immobilized MN gp120 (derived from HIV-1 subtype B strain MN) substrates (Fig. 1a). By contrast, CD4^‒^α4β7^+^ Jurkat T cells showed strong adhesion to gp120 upon inducing α4β7 activation by 0.5 mM Mn^2+^ and the adhesion was fully blocked by integrin α4β7 blocking antibodies Act-1 and FIB504 (Fig. 1a). In addition, the binding of soluble gp120 protein to CD4^‒^ Jurkat T cells and CD4^‒^α4β7^+^ Jurkat T cells showed consistent results (Supplementary Fig. S1b). These data indicate that HIV-1 envelope protein gp120 binds to the activated integrin α4β7 on the Jurkat T cells independent of CD4.

**Fig. 1:**
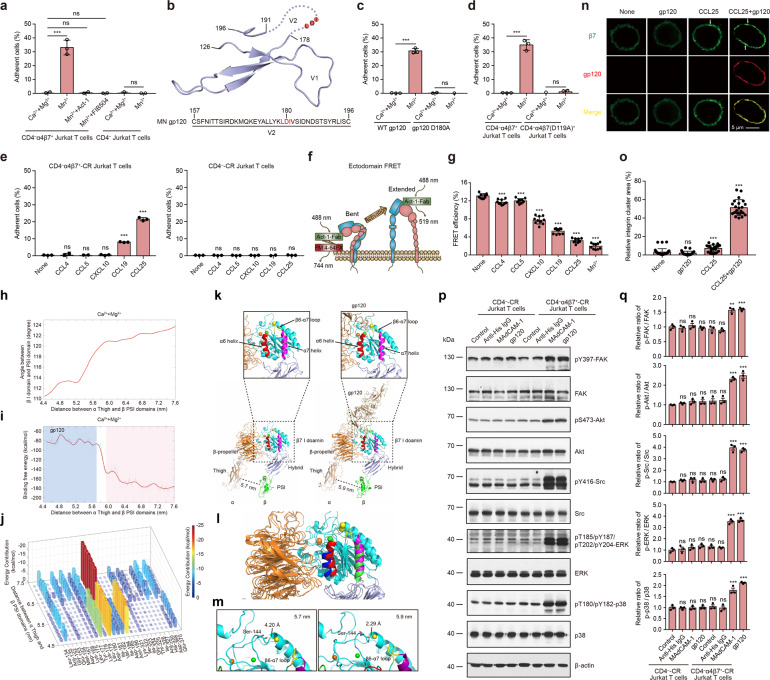
Distinct chemokines selectively induce HIV-1 gp120-integrin α4β7 binding via triggering conformer-specific activation of α4β7. **a** Adhesion of CD4^‒^ Jurkat T cells or CD4^‒^α4β7^+^ Jurkat T cells to immobilized gp120 in 1 mM Ca^2+^/Mg^2+^ or 0.5 mM Mn^2+^. **b** A diagram of the V1 and V2 domains of gp120 was drawn based on the crystal structure of BG505 SOSIP.664 gp120 (PDB: 3J5M). Light red shadow indicates integrin α4β7 binding site in the V2 loop. Dashed lines indicate the location of gp120 V2 loop. The sequence of the V2 domain of MN gp120 was provided at the bottom and the potential integrin α4β7 binding motif in gp120 is highlighted in red. **c** Adhesion of CD4^‒^α4β7^+^ Jurkat T cells to the immobilized gp120 or gp120 D180A in 1 mM Ca^2+^/Mg^2+^ or 0.5 mM Mn^2+^. **d** Adhesion of CD4^‒^α4β7^+^ Jurkat T cells and CD4^‒^α4β7(D119A)^+^ Jurkat T cells to immobilized gp120 in 1 mM Ca^2+^/Mg^2+^ or 0.5 mM Mn^2+^. **e** Adhesion of CD4^‒^-CR Jurkat T cells or CD4^‒^α4β7^+^-CR Jurkat T cells to the immobilized gp120 in 1 mM Ca^2+^/Mg^2+^ with and without chemokine stimulation. **f** Experiment setup for measuring FRET efficiency between integrin α4β7 β I domain and the plasma membrane (Ectodomain FRET). A composite of all molecules used is depicted. **g** FRET efficiency of CD4^‒^α4β7^+^-CR Jurkat T cells before and after treatment with 0.5 μg/ml chemokines or 0.5 mM Mn^2+^. **h** Relationship of the distance between integrin α Thigh and β PSI domains and the angle between β I domain and PSI domain in Ca^2+^/Mg^2+^. **i** Binding free energy profiles of integrin α4β7 headpiece to gp120 in Ca^2+^/Mg^2+^ during the conformational transition. **j** Per-residue free energy decomposition of the residues at the interface of integrin α4β7 headpiece and gp120 complex from the MM/GBSA. The residues of gp120 with energy contributions stronger than −1 kcal/mol along the conformational path of α4β7 headpiece are illustrated. The color bar is set in the range of −25–0 kcal/mol. **k** Snapshot of integrin α4β7 headpiece with a distance of 5.7 or 5.9 nm between α4 Thigh and β7 PSI domains. The α6 and α7 helices of the β7 I domain are colored in red and purple, and SyMBS, MIDAS, and ADMIDAS metal ions are colored in orange, green, and yellow spheres, respectively. **l** Superposition of integrin α4β7 headpiece with a distance of 5.7 or 5.9 nm between α4 Thigh and β7 PSI domains. The α6 and α7 helices of the β7 I domain were shown in red and purple in 5.7 nm structure, and in blue and green in 5.9 nm structure. **m** The change of the distance of ADMIDAS metal ion to the backbone carbonyl of Ser-144 located at β1-α1 loop region and the movement of the β6-α7 loop (yellow colored) during the transition from 5.7 to 5.9 nm between α4 Thigh and β7 PSI domains. **n** Confocal microscopy visualization of the integrin clustering on the plasma membrane of CD4^‒^α4β7^+^-CR Jurkat T cells. Integrin β7, green; gp120, red. White arrowheads indicate the representative integrin clusters. Scale bar, 5 μm. **o** The relative integrin cluster area was calculated as the percentage of the fluorescence intensity of integrin clusters in relation to that of the entire cell surface. **p**, **q** CD4^‒^-CR Jurkat T cells or CD4^‒^α4β7^+^-CR Jurkat T cells were pre-treated with CCL25 (0.5 μg/ml, in HBS with 1 mM Ca^2+^/Mg^2+^) for 15 min at room temperature. Then cells were stimulated with anti-His IgG (100 μg/ml), MAdCAM-1 (100 μg/ml) or gp120 (500 μg/ml) for 30 min at 37 °C, respectively. The expression and phosphorylation of FAK, Akt, Src, ERK, and p38 were determined by immunoblot analysis (**p**). The relative ratios of p-FAK/FAK, p-Akt/Akt, p-Src/Src, p-ERK/ERK, and p-p38/p38 were normalized to the values of CD4^‒^-CR Jurkat T cells without stimulation (Control) (**q**)

Integrin α4β7 binds to its natural ligand MAdCAM-1 through a critical interaction between the divalent cation in the metal ion-dependent adhesion site (MIDAS) located in β7 I domain and the Asp residue in the LDT motif in MAdCAM-1. The metal ion in MIDAS directly coordinates the side chain of the acidic residue characteristic of all integrin ligands. A similar LDI motif at the position 179–181 (numbering for HIV-1 clone HXB2) is conserved in the V2 loop of gp120 (Fig. 1b). To test whether β7 MIDAS and gp120 LDI motif mediate the α4β7-gp120 interaction, we mutated Asp180 in gp120 LDI motif to Ala (gp120 D180A). The D180A mutation completely abolished the adhesion of CD4^‒^α4β7^+^ Jurkat T cells to the immobilized gp120 (Fig. 1c) and the binding of soluble gp120 protein to CD4^‒^α4β7^+^ Jurkat T cells in 0.5 mM Mn^2+^ (Supplementary Fig. S2a), indicating the essential role of Asp180 residue in gp120 in mediating α4β7-gp120 interaction. Moreover, the critical MIDAS divalent cation coordinating residues D119 in β7 I domain was mutated to Ala to abolish the metal ion in MIDAS, and β7 D119A mutant was stably expressed in CD4^‒^ Jurkat T cells (CD4^‒^α4β7(D119A)^+^ Jurkat T cells) at a similar level of α4β7 in CD4^‒^α4β7^+^ Jurkat T cells (Supplementary Fig. S2b). D119A mutation completely abolished the adhesion of CD4^‒^α4β7^+^ Jurkat T cells to gp120 (Fig. 1d) and the binding of soluble gp120 protein to those cells in 0.5 mM Mn^2+^ (Supplementary Fig. S2c). Collectively, these data indicate that the divalent cation in integrin β7 MIDAS and Asp180 in gp120 LDI motif mediate the critical interaction between integrin α4β7 and gp120.

Chemokines are major stimuli that induce integrin α4β7 activation in the gut. We next examined the regulation of gp120 binding to α4β7 by gut-expressing chemokines, including CCL4, CCL5, CCL19, CCL25, and CXCL10, which are upregulated in the GALT of SIV-1 infected macaques. The CCR5 (CCL4, CCL5 receptor) and CCR7 (CCL19 receptor) were endogenously expressed in Jurkat T cells. Because Jurkat T cells do not have endogenous receptors for CCL25 and CXCL10, we generated CD4^‒^α4β7^+^ and CD4^‒^ Jurkat T cells that stably expressed receptors for these chemokines (CD4^‒^α4β7^+^-CR and CD4^‒^-CR Jurkat T cells) (Supplementary Fig. S3). CCL19 or CCL25 stimulation significantly increased the binding of gp120 protein to CD4^‒^α4β7^+^-CR Jurkat T cells (Fig. 1e and Supplementary Fig. S4). By contrast, CCL4, CCL5, and CXCL10 failed to promote the binding of gp120 to these cells although these chemokines can induce α4β7 activation and promote α4β7 adhesion to VCAM-1 (Supplementary Fig. S5). These data indicate that the gp120 only binds to certain active conformers of integrin α4β7 induced by CCL19 and CCL25.

Different chemokines have been shown to induce distinct active states of integrin α4β7, which is associated with global conformational rearrangement of integrin ectodomains from a low-affinity, bent (close) conformation to an high-affinity, extended (open) conformation. We next used fluorescence resonance energy transfer (FRET) assay to examine the extension of α4β7 ectodomains. To assess the orientation of integrin α4β7 ectodomain relative to the plasma membrane, integrin α4β7 headpiece was labeled with Alexa Fluor 488-conjugated Act-1 Fab fragment, which binds to β7 I domain as donor, and the plasma membrane was labeled with FM 4–64FX as acceptor (Fig. 1f). Compared with the unstimulated CD4^‒^α4β7^+^-CR Jurkat T cells, cells treated with chemokines or Mn^2+^ showed significantly lower FRET efficiency, indicating the extension of α4β7 ectodomain and the activation of α4β7 (Fig. 1g). Notably, CCL19, CCL25, and Mn^2+^ induced much lower FRET signals than other chemokines, indicating CCL19, CCL25, and Mn^2+^ induced the highly extended active conformations of α4β7, while CCL4, CCL5, and CXCL10 induced less extended active conformers of α4β7.

To further investigate the correlation between different active conformers of α4β7 and their binding affinities for gp120, we applied Molecular Dynamics (MD) simulations to study the binding free energy between gp120 and α4β7 headpiece during its transition from the bent to extended conformation. The homology model of the five-domain headpiece of α4β7 containing the β-propeller and Thigh domains (residues 1–586) of the α4-subunit and the β I, hybrid and PSI domains (residue 41–503) of the β7-subunit was constructed. Because the structure of MN gp120 has not been solved, the initial structural conformation of the MN gp120 with fully glycosylated was homology modeled from the fully glycosylated HIV-1 envelope glycoprotein trimer JR-FL (PDB: 5FYK), which shares 87% sequence identity with MN gp120 (Supplementary Fig. S6). The molecular mechanics/generalized born surface area (MM/GBSA) method was applied to estimate the binding affinity of the MN gp120 to the α4β7 headpiece. The distance between the center of mass of the α4 Thigh and β7 PSI/hybrid domain was used to define the conformational changes during integrin activation. During the transition of the gp120 bound α4β7 headpieces from the bent to extended conformation in Ca^2+^/Mg^2+^ condition, which are associated with a 4.4–7.6 nm separation between the Thigh and PSI domain and the hybrid swing-out with an increase of angle between the β I domain and PSI domain during integrin conformational transition (Fig. 1h). The binding free energy profiles showed a quick increment of the binding affinity when the distance between α4 Thigh and β7 PSI domains was increased from 5.7 to 5.9 nm (Fig. 1i), indicating α4β7 displays a strong binding affinity to gp120 only after it extend to certain stage. This data is consistent with the FRET results that only highly extended active conformers of α4β7 showed strong binding to gp120. Per-residue energy decomposition analysis of gp120 from the MM/GBSA binding free energy evaluation suggest that the major integrin binding interface in gp120 is located at a segment (residues 167–186) in V2 domain (Fig. 1j). Among these residues, D180 had a significant role in stabilizing the gp120-α4β7 interaction, which is consistent with the results that D180A mutation abolished the gp120-α4β7 interaction (Fig. 1c and Supplementary Fig. S2a). Comparing the critical transition of α4β7 headpiece from 5.7 to 5.9 nm revealed a perceptible downward shift of α7 helix in β7 I domain. The center of mass (COM) of α7 helix moved downward about 1.86 Å and a tilt movement of the α6 helix was also observed with ~11.24° shift between the two conformations (Fig. 1k, l). During this transition, the ADMIDAS metal ion shifted more closer towards the backbone carbonyl of Ser-144 located at β1-α1 loop region from 4.20 to 2.29 Å (Fig. 1m). Moreover, the movement of the ADMIDAS metal ion was also accompanied with the arrangement of the β6-α7 loop (Fig. 1m). These conformational rearrangements in β7 I domain indicate direct correlations with integrin activation.

The ligand binding to integrin can induce the clustering of integrins on the plasma membrane and trigger the activation of intracellular signaling pathways. To investigate the effect of gp120-α4β7 interaction on integrin downstream signaling, we firstly studied the gp120-induced α4β7 clustering on the plasma membrane (Fig. 1n, o). Compared with untreated CD4^‒^α4β7^+^-CR Jurkat T cells, CCL25 stimulation induced weak clustering of α4β7 on the cell surface due to the chemokine-induced activation of α4β7.^4^ Pretreatment of cells with Alexa 568-conjuncted gp120 along did not induce α4β7 clustering because gp120 did not bind to inactive α4β7. Notably, the α4β7 clustering signal was significantly increased by addition of gp120 after CCL25 treatment along with the extensive colocalization of α4β7 with gp120, indicating the binding of gp120 to the activated α4β7 efficiently promoted the clustering of α4β7.

Next, we examined the effect of gp120-α4β7 binding on the major downstream signal pathways of integrin, including FAK, Akt, Src, ERK, and p38 (Fig. 1p, q). CCL25-treated CD4^‒^α4β7^+^-CR Jurkat T cells were incubated with gp120 or MAdCAM-1 at 37 °C for 30 min, and then the expression and phosphorylation of FAK, Akt, Src, ERK, and p38 were determined. Gp120 and MAdCAM-1 induced similar increases in the phosphorylation of all kinases, indicating the activation of multiple integrin downstream signals. Thus, gp120 can trigger α4β7 outside-in signaling in a manner similar to its natural ligand MAdCAM-1. As a control, gp120 and MAdCAM-1 failed to induce the activation of these signal pathways in CD4^‒^-CR Jurkat T cells, which lack α4β7.

To further confirm the gp120-α4β7 interaction and its related functions using a more physiologically relevant form of the HIV envelope protein, we expressed CCR5-tropic BG505 SOSIP.664 gp140 trimers, which contain three gp120 and gp41 ectodomain subunits. Consistent with the results of monomeric MN gp120, the binding of CD4^‒^α4β7^+^ Jurkat T cells to the immobilized and soluble BG505 SOSIP.664 was significantly enhanced by 0.5 mM Mn^2+^ (Supplementary Fig. S7a, b). Furthermore, CCL19 or CCL25 stimulation significantly increased the binding of BG505 SOSIP.664 to CD4^‒^α4β7^+^-CR Jurkat T cells (Supplementary Fig. S7c, d) and activated integrin downstream signals (Supplementary Fig. S8). Therefore, monomeric MN gp120 and trimeric BG505 SOSIP.664 exhibited similar binding to integrin α4β7 and triggered similar activation of integrin downstream signals.

It’s reported that α4β7 and CCR5 formed complexes on γδT cells in the absence of CD4 and gp120 binding to T cells was partially inhibited when CCR5 function was inhibited. In addition, integrin α4β7 was colocalized with CD4 and CCR5 on α4β7^high^ CD4^+^ T cells.^1^ These findings suggest CCR5 contributes to the gp120-α4β7 interaction. However, our data showed that knock-out CCR5 in CD4^‒^α4β7^+^ Jurkat T cells did not affect gp120 binding to the cells (Supplementary Fig. S9a, b), suggesting that CCR5 is not necessary for efficient gp120-α4β7 interaction when α4β7 is highly activated. Similar results were obtained when using BG505 SOSIP.664 proteins (Supplementary Fig. S9c).

Th17 cells play a critical role in the immune defense of the gut mucosa. Th17 cells are preferentially depleted from the GALT of HIV-infected individuals with rapid disease progression. The depletion of Th17 in the gut could compromise the integrity of the gut mucosal barrier. It is noteworthy that these cells express both integrin α4β7 and CCL25 receptor CCR9. CCL25 is expressed by epithelial cells in the small intestine, especially those in the crypt region most closely associated with MAdCAM-1-expressing vessels. According to our findings, CCL25 can efficiently promote gp120-α4β7 binding, thus it is tempting to speculate that CCL25 may enhance the infection of Th17 cells by HIV through this mechanism.

CCL19-CCR7 axis is another agonist for α4β7-gp120 binding. Both naïve T cells and central memory T cells (T_CM_) express CCR7. Moreover, gut-homing naïve T cells and T_CM_ also express integrin α4β7. Colon and small intestine express CCL19, which is further upregulated in the inflamed intestine. Because CCL19 can significantly promote α4β7-gp120 binding, CCL19-CCR7 axis could enhance the infection of naïve T cells and T_CM_ cells in the gut during HIV infection. Indeed, a significant reduction in naïve T cell number and the impaired function of these cells are associated with HIV-1 infection. Moreover, T_CM_ cells are considered to be the most important reservoir of latent HIV. CCL19 has the potential to activate integrin α4β7 thus promotes the entry of HIV-1 into T_CM_ cells.

Our data showed that the binding of gp120 to α4β7 on T cells activated multiple signaling pathways in T cells, including FAK, Akt, Src, ERK, and p38. Some of these signaling pathways are closely related to HIV replication and depletion of CD4 T cells. Firstly, HIV-1 utilizes ERK and p38 pathways to produce new virions. Secondly, p38 activation is required for the gp120-mediated apoptosis observed in primary human T cells during HIV-1 infection. Thirdly, it is reported that Akt pathway plays a role in HIV-1 reservoir formation and blocking Akt activation limits HIV-1 recovery from latently infected T cells. Therefore, gp120-α4β7 binding-induced integrin downstream signaling has an important role in HIV-1 infection and virus replication. Akt, ERK, and p38 pathways could be potential targets for anti-HIV drug discovery.

In summary, our study demonstrates that different gut-expressing chemokines selectively promote gp120-α4β7 interaction by inducing conformer-specific activation of α4β7. Gp120 bound to α4β7 active conformers with highly extended conformation induced by CCL19 and CCL25 and activated multiple intracellular pathways. Thus, only particular chemokines that can induce the active α4β7 conformers with ectodomains extended beyond a certain degree may promote HIV infection of T cells. Specific chemokine receptors might be potential drug target for treatment of HIV infection.

## Supplementary information

Supplemental Material-SIGTRANS-02671R

## Data Availability

The data used and analyzed in this study are available in the main text and the Supplementary Materials. Any other raw data that support the findings of this study are available from the corresponding author upon reasonable request.
