# Supplementary material for: Distinct chemokines selectively induce HIV-1 gp120-integrin α4β7 binding via triggering conformer-specific activation of α4β7
Source: Signal Transduct Target Ther. 2021 Jul 16;6:265. doi: 10.1038/s41392-021-00582-8 (PMC8282615; doi:10.1038/s41392-021-00582-8)
Supplement: Supplementary file 1 — Supplemental Material-SIGTRANS-02671R [file 41392_2021_582_MOESM1_ESM.docx]

**Supplementary Materials for**

**Distinct chemokines selectively induce HIV-1 gp120-integrin α4β7 binding via triggering conformer-specific activation of α4β7**

Shu Wang^1^, ChangDong Lin^1^, Yue Li^1^, ZhaoYuan Liu^1^, JunLei Wang^1^, YouHua Zhang^1^, ZhanJun Yan^2^, YueBin Zhang^3^, GuoHui Li^3^, JianFeng Chen^1,4^

Correspondence:

YueBin Zhang (zhangyb@dicp.ac.cn) or GuoHui Li ([ghli@dicp.ac.cn](mailto:ghli@dicp.ac.cn)) or JianFeng Chen (jfchen@sibcb.ac.cn)

**This PDF file includes:**

Materials and Methods

Figures S1 to S10

**Materials and Methods**

**Antibodies and reagents**

Human CCL19 and CCL25 were from R&D Systems. Human CCL4, CCL5 and CXCL10 were from Novoprotein. The antibodies used were anti-human α4β7 (Act-1), anti-human CCR9 (557975; BD), anti-human CXCR3 (CD183; 550633; BD), anti-human CD4 (RPA-T4; 14-0049; eBioscience), anti-human CCR5 (ab11466; abcam), anti-FAK (610088; BD; 1:1000), anti-phosphorylated FAK at Tyr397 (611723; BD; 1:1000); anti-Akt (4685; CST; 1:1000); anti-phosphorylated Akt at Ser473 (4085; CST; 1:1000); anti-Src (2109; CST; 1:2000); anti-phosphorylated Src at Tyr416 (2101S; CST; 1:1000); anti-ERK (ab17942; Abcam; 1:1000); anti-phosphorylated ERK at Thr180/Tyr187/Thr202/Tyr204 (ab50011; Abcam; 1:10000); anti-p38 MAPK (8690; CST; 1:2000), anti-phosphorylated p38 MAPK at Thr180/Tyr182 (05-1059, Millipore, 1:5000). Rat mAb FIB504 against human/mouse β7 and mouse mAb AIIB2 against human/mouse β1 were prepared by using hybridomas (Developmental Studies Hybridoma Bank, University of Iowa). FITC-conjugated goat anti-rat IgG (629511), and FITC-conjugated goat anti-mouse IgG (626511) were from Invitrogen. Alexa Fluor APC-conjugated goat anti-mouse IgG (405322) was from Biolegend.

**cDNA Construction and Cell lines**

cDNAs of human α4 and β7 subunits were constructed in vector pCDH-puro (Invitrogen). The β7 subunit with MIDAS mutation D119A was generated using QuikChange (Stratagene). Human CD4 and chemokine receptor CCR5 single-guide RNA (sgRNA) designed by GN20GG rule were constructed into vector lentiCRISPR v2 (Addgene). The 20-nucleotide sgRNA sequence: 5′-GTCTGTAAAACGGGTTACCC-3′ for the *CD4 gene*, 5′-GGTCCTGCCGCTGCTTGTCA-3′ for the *CCR5 gene*. Jurkat T cells stably expressing CXCR3 and CCR9 (CD4^‒^-CR Jurkat T cells and CD4^‒^α4β7^+^-CR Jurkat T cells) were generated by the electroporation of cDNAs of CXCR3 and CCR9 followed by selection using puromycin (2 μg/ml).

**Flow Cytometry**

Cell surface expression of integrin α4β7 and CD4 in Jurkat T cells was determined by staining with mAbs Act-1 and RPA-T4, and then measured using a FACSCelesta^TM^ (BD Biosciences) and analyzed using FlowJo software.

**Protein expression and purification**

Codon optimized MN gp120 sequence was kindly provided by Prof. Brian Seed and Dr. Slim Sassi (Massachusetts General Hospital, Boston, USA). His tagged gp120 at C-terminus was constructed into vector pcDNA3.1-hygro. Gp120 expressing construct was transiently transfected into HEK293T cells (ATCC) and cultured in DMEM supplemented with 10% fetal bovine serum (Invitrogen). The protein was purified by Ni-NTA (Qiagen) affinity chromatography according to the manufacturer’s instructions.

The plasmids expressing BG505 SOSIP.664, Furin and PGT145 were kindly provided by Prof. Lu Lu and Dr. Miao Cao (Fudan University, Shanghai, China). The BG505 SOSIP.664 construct was co-transfected with the plasmid expressing Furin (4:1) in HEK293T cells. Transfection supernatants were harvested after 7 days. BG505 SOSIP.664 gp140 trimers were purified by affinity chromatography using a PGT145 column. The PGT145 column was made from CNBr-activated Sepharose 4B beads (GE Healthcare) coupled to the bNAb PGT145.

**Static cell adhesion assay**

Briefly, 96-well plates were coated with 100 µg/ml gp120 or 50 µg/ml BG505 SOSIP.664 overnight and blocked with 1% BSA for 1 h at 37°C, BCECF labelled cells were treated with distinct chemokines (0.5 μg/ml) or 0.5 mM Mn^2+^ and incubated in wells for 10 minutes, nonadherent cells removed by serial washes. The number of adherent cells remaining, expressed as a percentage of the total number of labeled cells, was determined with a fluorescent plate reader (Synergy HT, BioTek Instruments).

**Soluble ligand binding assay**

Jurkat T cells were diluted in HBS (20 mM HEPES, 150 mM NaCl, pH 7.4) containing different divalent cations (1 mM Ca^2+^/Mg^2+^ for unstimulated and chemokine-treated conditions and 0.5 mM Mn^2+^ for Mn^2+^-treated condition). Cells before and after soluble chemokine (0.5 μg/ml) or Mn^2+^ stimulation were fixed with paraformaldehyde (2 %). Recombinant gp120-His (500 μg/ml), MAdCAM-1-His (50 μg/ml), VCAM-1-His (50 μg/ml) or BG505 SOSIP.664-His (100 μg/ml), was pre-incubated with mouse anti-His mAb and APC-conjugated goat anti-mouse IgG in HBS containing 1 mM Ca^2+^/Mg^2+^ or 0.5 mM Mn^2^ and then incubated with cells for 30 min at room temperature. α4β1-VCAM-1 interaction was disrupted by pretreating the cells with β1 blocking antibody AIIB2 (20 μg/ml) for 5 min at 37 °C before addition of VCAM-1-His mixture. Cells were washed twice and measured using a FACSCelesta^TM^ (BD Biosciences) and analyzed using FlowJo software.

**FRET assay**

For detecting the orientation of integrin ectodomain relative to cell membrane, cells before and after soluble chemokine (0.5 μg/ml) stimulation were seeded on poly-L-Lysine (100 μg/ml) coated surface in HBS containing 1 mM Ca^2+^/Mg^2+^ for 30 min at 37 ̊C, 0.5 mM Mn^2+^ was added to activate integrin where indicated. Adherent cells were fixed with 3.7% paraformaldehyde for 15 min at room temperature and non-specific sites were blocked by incubation with 10% serum rich medium for 10 min at room temperature. Then cells were stained with 10 μg/mL Alexa Fluor 488-conjugated Act-1 Fab fragment for 30 min at 37 ̊C. After two washes, cells were labeled with 10 μM FM 4-64FX (Invitrogen) for 1 min on ice, washed once, immediately mounted with Mowiol 4-88 (Polysciences Inc.) mounting solution under a coverslip. The mounted slides were kept in the dark and subjected to photobleach FRET acquisition by a confocal microscope (TCS SP8, Leica). FRET efficiency (E) was calculated as E = 1-(F_donor_(d)_Pre_/F_donor_(d)_Post_), where F_donor_(d)_Pre_ and F_donor_(d)_Post_ are the mean donor emission intensity of pre- and post-photobleaching.

**MD simulations**

The initial MN gp120 conformation was homology modeled from the full glycosylated HIV-1-envelope (Env) trimer (PDB: 5FYK) using MODELLER package. The missing residues of the template was modeled as short loops in the MN gp120 model. The glycosylation patterns of the MN gp120 was also obtained from the full glycosylated template. 17 N-linked glycosylation sites were modeled and 9 disulfide bridges were formed using psfgen package in VMD. The CHARMM36 force fields for protein and glycans as well as ions were implemented and the TIP3P water molecules were used to solvate the glycosylated MN gp120 model into a rectangle box. Then, 0.1M NaCl was used to neutralize the system, yielding the MN gp120 MD simulation system containing a total of ~120,596 atoms with an initial dimension of 10nm x 8.8nm x 14.5nm.

Gaussian accelerated MD simulation is a variant of accelerated MD which adds the boost potential following Gaussian distribution to smoothen the system potential energy surface (PES) to decrease the energy barriers and accelerate the transition between different meta-stable states of the system, thus, enable unconstrained enhanced sampling to probe much broader conformational space. The integrin α4β7 binds to LDI motif in the V2 region of MN gp120. The MIDAS Mg^2+^ directly coordinated the Asp side chain of the ligand, which is occluded in both 5FYK template and MN gp120 model. Hence, Gaussian accelerated MD (GaMD) simulations, which enable unconstrained enhanced sampling to probe much broader conformation space, were conducted to identify the possible solvent-exposed conformation in the LDI region of MN gp120 (Supplementary Fig. S10). The LDI tripeptide was solvent-accessible after 4×800ns GaMD simulations. Prior to performing Gaussian accelerated MD, classical MD equilibrations were conducted. The system was heated to 300K with harmonic position restraints of backbone atoms in the isothermal-isobaric ensemble (NPT) and the restraint was set to 10 kcal/mol・Å2. Subsequently, the restraints were released and 10 ns NPT simulations were used to equilibrate the density of the system. The bond lengths involving hydrogen atoms were constrained using the SHAKE algorithm, enabling the 2 fs time step. The Langevin dynamics with a collision frequency of 5 ps were using the coupling the temperature of the system at 300K and the Berendsen barostat was used to control the pressure of the system at 1 atm with a relaxation time of 2ps. All simulations were performed with GPU version of AMBER 18 package. The equilibrated system was further used as starting point for GaMD simulation in canonical ensemble (NVT). In the GaMD simulations, dual-boost scheme was applied, in which two acceleration potentials (the torsional potential and the total potential) were exerted simultaneously to the system and the system threshold energy was set to E=Vmax. 4 individual GaMD trajectories were generated including 10 ns pre-equilibration classical MD following by 50ns GaMD equilibration and 800 ns GaMD production.

The snapshot of MN gp120 after GaMD simulation was used to build the initial state of gp120-α4β7 complex. The five-domain α4β7 headpiece conformations in the closed and open states were obtained from our previous work. The gp120-α4β7 complex was separated 5 nm away from each other initially and the principal axes of gp120 was set to be parallel to the ligand binding groove formed by the interface between α4 and β7. The TIP3P water molecules were used to solvate the complex into a rectangle box and 0.1 M NaCl was used to neutralize the system, which yields a total of ~461,489 atoms in the closed headpiece and ~460,709 atoms in the open headpiece systems, respectively. Similar heating and equilibration processes were carried out to that of gp120 simulation system. After the equilibration, the constant velocity steered molecular dynamics (SMD) simulation was used, which is implemented using NAMD, to pull the D180 approaching the MIDAS Mg^2+^ ion of β7 within 50 ns simulation. During the SMD simulation, the heavy atom positions of the α4β7 were harmonic restrained with a spring constant of 5 kcal/mol・Å2 and the secondary structure elements of gp120 were also restrained using SSRestraints Plugin in NAMD. Finally, 12 individual GaMD trajectories were generated including 10 ns pre-equilibration classical MD following by 50 ns GaMD equilibration and 800 ns GaMD production in the closed and open headpiece conformations of α4β7 binding with gp120, respectively. The positions of α4 Thigh and β7 PSI domains were restrained during the GaMD simulations to maintain the corresponding closed and open states of the headpiece. The partial nudged elastic band (PNEB), all C-alpha atoms of gp120 and α4β7 headpiece, were used to explore the minimum energy path between closed and open states when gp120 is bound. The PNEB describes a path for conformational change by identifying a series of discrete intermediate states (also named as images) and a simultaneous energy minimization of the total system was performed to find the minimum energy path while keeping the endpoint images fixed. The two possible stable binding endpoints to gp120 in the closed and open α4β7 headpieces were filtered using MM/GBSA energy evaluation based on the GaMD simulations in previous step. 16 images connecting the closed and open headpieces were used with a fixed spring constant of 2 kcal/mol・Å2 and the binding energies between gp120 and intermediate images were estimated using MM/GBSA method after 15 ns NEB MD simulations of each image. The PNEB routines were implemented in the pmemd module of the Amber 18 package and the parallel-MPI simulations were performed with GPU processors.

**Confocal microscopy**

Cover glasses were coated with poly-L-Lysine (100 μg/ml), Cells with or without soluble chemokine (0.5 μg/ml) stimulation were fixed with paraformaldehyde (2 %) then incubated on the cover glass for 20 min at RT and blocked by 10% FBS. For gp120-treated cells, CD4^‒^α4β7^+^-CR Jurkat T cells with or without soluble chemokine (0.5 μg/ml) stimulation were treated for 30 min at 37℃ with 568-conjuncted gp120 (500 μg/ml), anti-β7 mAb FIB27 (5 μg/ml) was used to stain β7 at room temperature for 2 hours, followed by FITC-conjugated goat anti-rat IgG (H + L).

**Measurement of integrin clustering**

β7 fluorescence was acquired with identical excitation and exposure settings using a Plan Neofluar 63× NA 1.4 oil-immersion objective on an inverted confocal microscope (model LSM510; Carl Zeiss Micro Imaging, Inc.), focusing on the glass coverslip by using the maximal reflection of the laser light. Intensity histograms of cells were obtained after smoothing (3 × 3 kernel), background subtraction, and manual selection of the cell surface using MetaMorph software (Molecular Devices) and exported to Excel (Microsoft) for further analysis. The relative area of integrin clusters in respect to the entire cell surface was obtained from fluorescence microscopy images of at least 20 cells per condition. Fluorescence images of β7 integrin fluorescence were obtained with a Plan Neo-Fluar 100× NA 1.45 objective mounted on an Axiovert 100M (both from Carl Zeiss MicroImaging, Inc.) equipped with a 12-bit digital charge-coupled device camera (model Orca 4742–95; Hamamatsu Photonics) controlled by the Openlab software (Improvision). Image analysis was performed after manually setting of the intensity threshold using MetaMorph software.

**Western blotting**

CD4^‒^-CR Jurkat T cells and CD4^‒^α4β7^+^-CR Jurkat T cells were diluted in HBS containing 1 mM Ca^2+^/Mg^2+^ before and after soluble CCL25 (0.5 μg/ml) stimulation. Then, 500 μg/ml gp120-His, 100 μg/ml MAdCAM-1-His or 50 μg/ml BG505 SOSIP.664-His was added to the mixture and incubated for 30 min at 37 ̊C. Cells were washed and lysed with lysis buffer (20 mM HEPES, pH 7.4, 150 mM NaCl, 1% Triton X-100, 0.05% Tween-20, Complete protease inhibitor cocktail tablets and PhosSTOP phosphatase inhibitor cocktail tablets) on ice for 30 minutes. Cell lysates were then analyzed by blotting for FAK, pY397-FAK, Akt, pS473-Akt, Src, pY416-Src, ERK, pT185/pY187/pT202/pY204-ERK, p38, pT180/pY182-p38 and β-actin. β-actin was detected by immune blot as a loading control. Intensity analyses were conducted using Image J software.

**QUANTIFICATION AND STATISTICAL ANALYSIS**

Statistical significance was determined by Student’s t test (GraphPad, version 7). The resulting p values are indicated as follows: ns, not significant; *, p < 0.05; **, p < 0.01; ***, p < 0.001. Data represent the mean ± SEM of at least three independent experiments.

**Figure S1.**

**
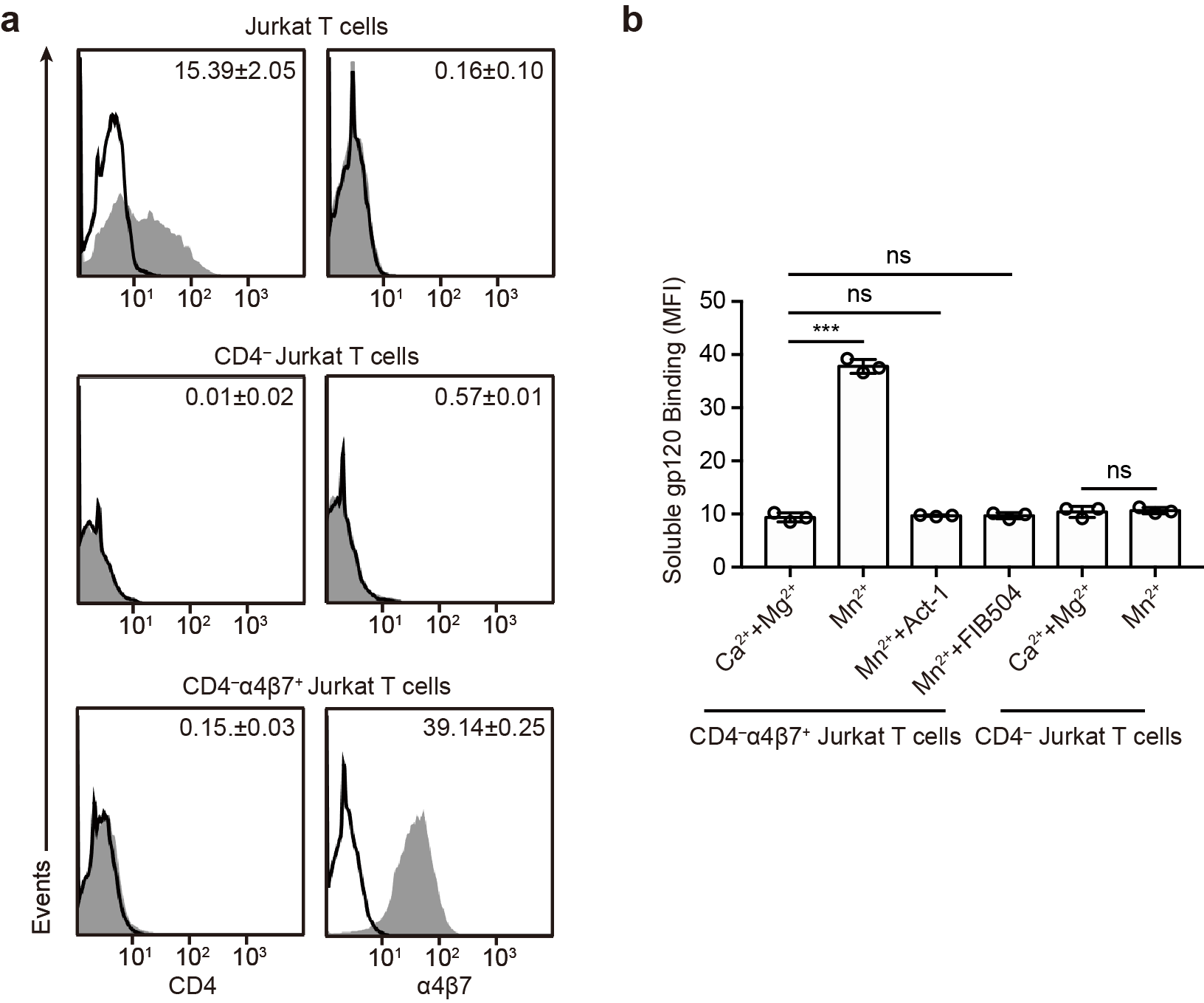
**

**Fig. S1 Gp120 binds to the activated integrin α4β7 independent of CD4.**

**a** Cell surface expression of CD4 and integrin α4β7 was determined by flow cytometry in Jurkat T cells (upper panels), CD4^‒^ Jurkat T cells (middle panels) and CD4^‒^α4β7^+^ Jurkat T cells (bottom panels). Numbers within the panel showed the specific mean fluorescence intensities. Opened histogram: mock control.

**b** Binding of soluble gp120 to CD4^‒^ Jurkat T cells or CD4^‒^α4β7^+^ Jurkat T cells was calculated with the specific mean fluorescence intensity (MFI) in 1mM Ca^2+^/Mg^2+^ or 0.5 mM Mn^2+^.

**Figure S2.**


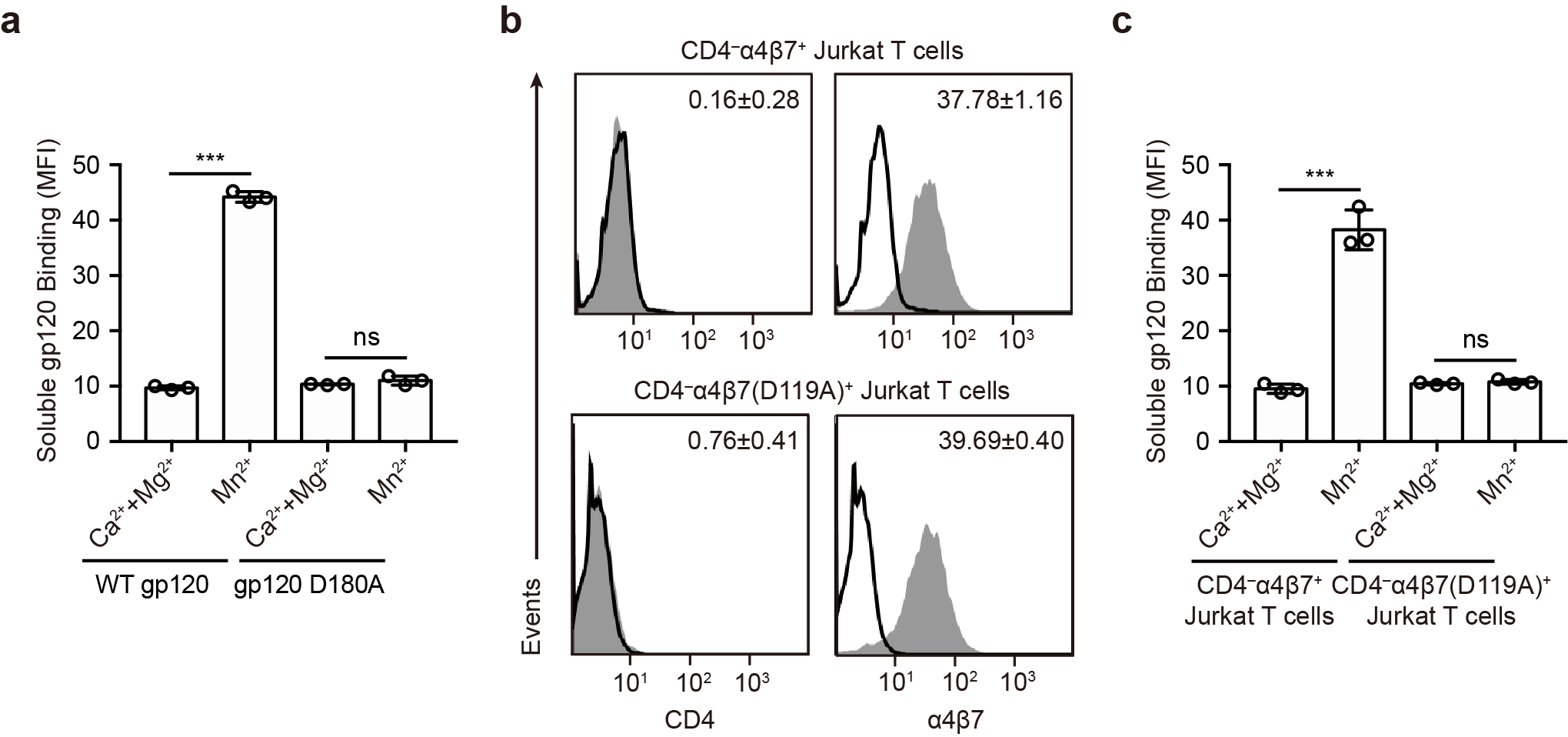


**Fig. S2 Gp120 LDI motif and integrin β7 MIDAS are critical for gp120-α4β7 interaction.**

**a** Binding of soluble WT gp120 or gp120 D180A to CD4^‒^α4β7^+^ Jurkat T cells was calculated with the specific mean fluorescence intensity (MFI) in 1mM Ca^2+^/Mg^2+^ or 0.5 mM Mn^2+^.

**b** Cell surface expression of CD4 and integrin α4β7 was determined by flow cytometry in CD4^‒^α4β7^+^ Jurkat T cells (upper) and CD4^‒^α4β7(D119A)^+^ Jurkat T cells (bottom). Numbers within the panel showed the specific mean fluorescence intensities. Opened histogram: mock control.

**c** Binding of soluble gp120 to CD4^‒^α4β7^+^ Jurkat T cells and CD4^‒^α4β7(D119A)^+^ Jurkat T cells was calculated with the specific mean fluorescence intensity (MFI) in 1mM Ca^2+^/Mg^2+^ or 0.5 mM Mn^2+^.

**Figure S3.**


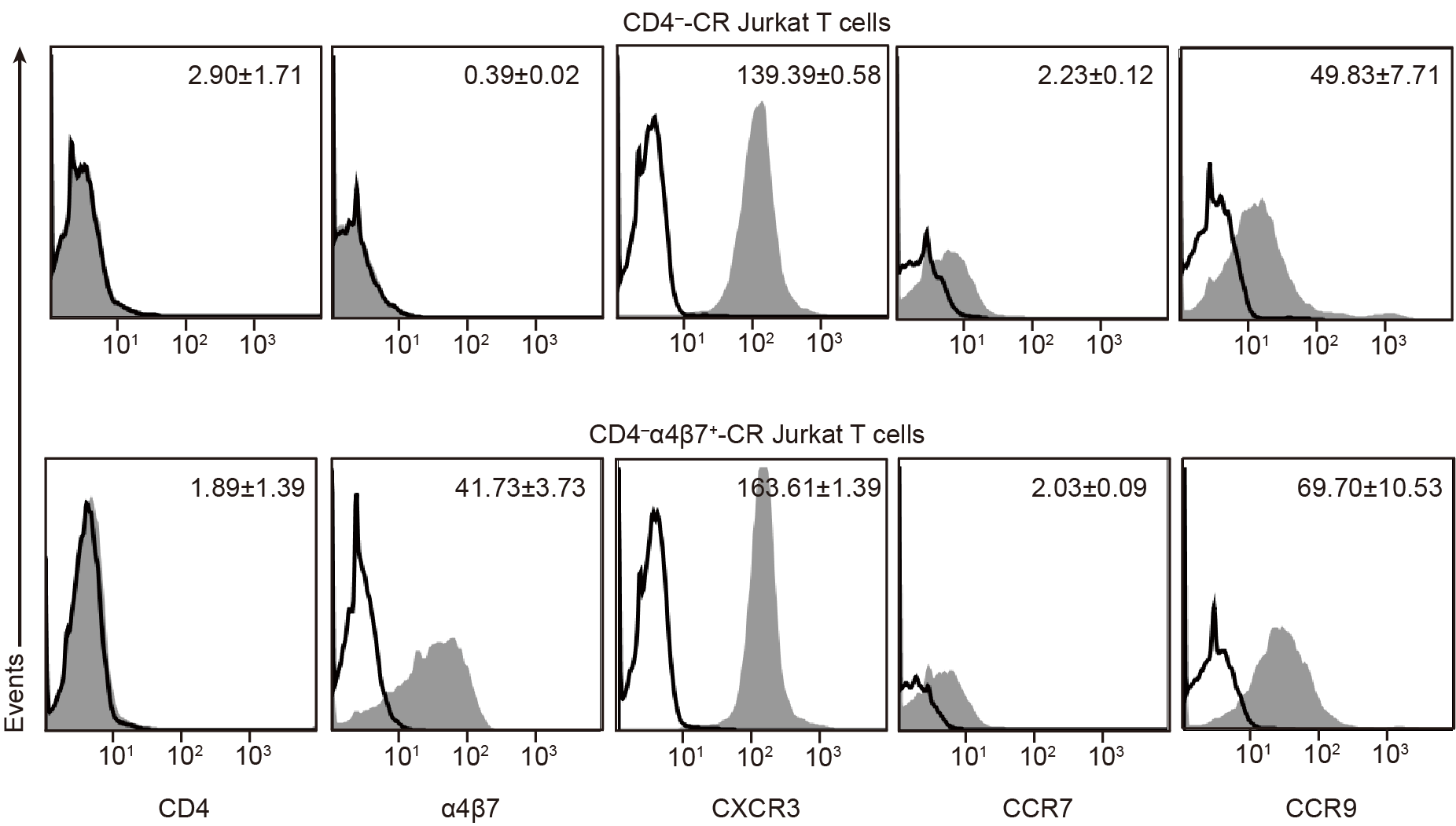


**Fig. S3 Cell surface expression of CD4, integrin α4β7 and chemokine receptors in Jurkat T cells.**

The expression of the indicated molecules was determined by flow cytometry. Numbers within the panel showed the specific mean fluorescence intensities. Opened histogram: mock control. CD4^‒^-CR Jurkat T cells, upper; CD4^‒^α4β7^+^-CR Jurkat T cells, bottom.

**Figure S4.**


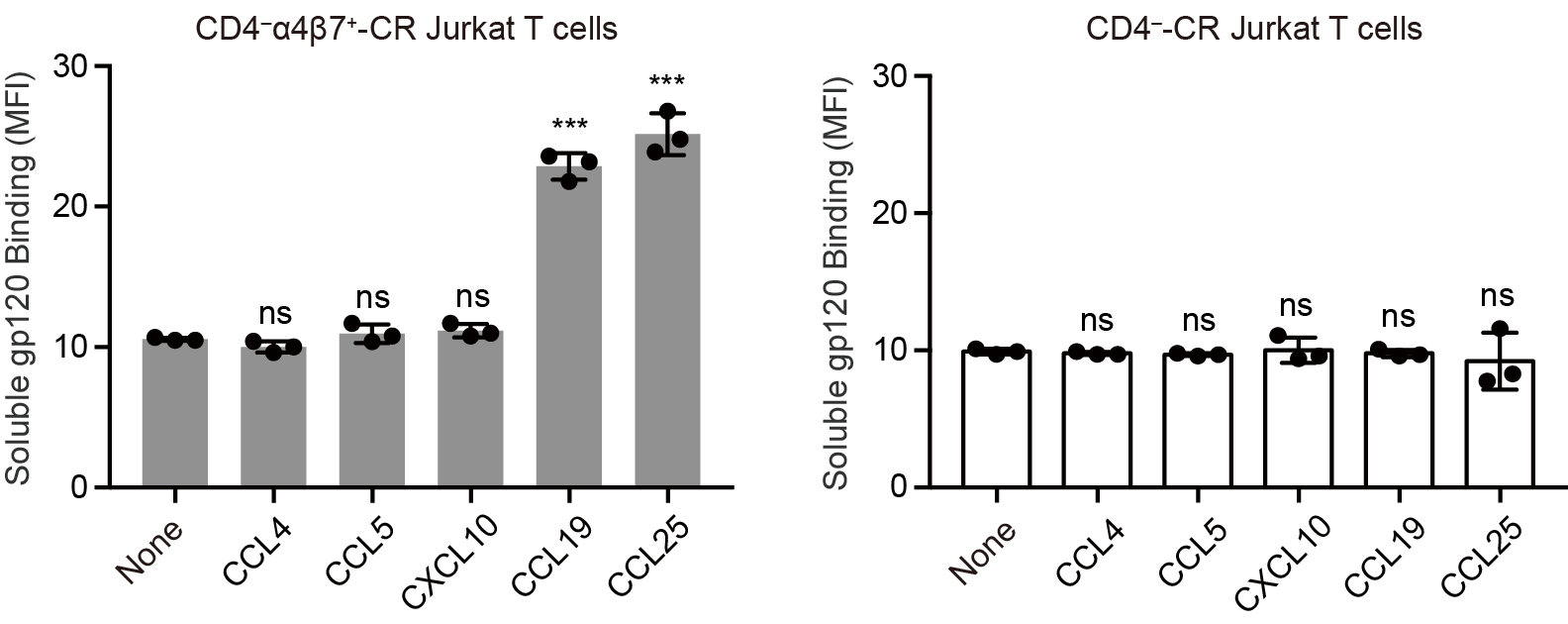


**Fig. S4 Regulation of integrin α4β7-gp120 binding by different chemokines.**

Binding of soluble gp120 to CD4^‒^-CR Jurkat T cells or CD4^‒^α4β7^+^-CR Jurkat T cells was calculated with the specific mean fluorescence intensity (MFI) in 1mM Ca^2+^/Mg^2+^ with and without chemokine stimulation.

**Figure S5.**


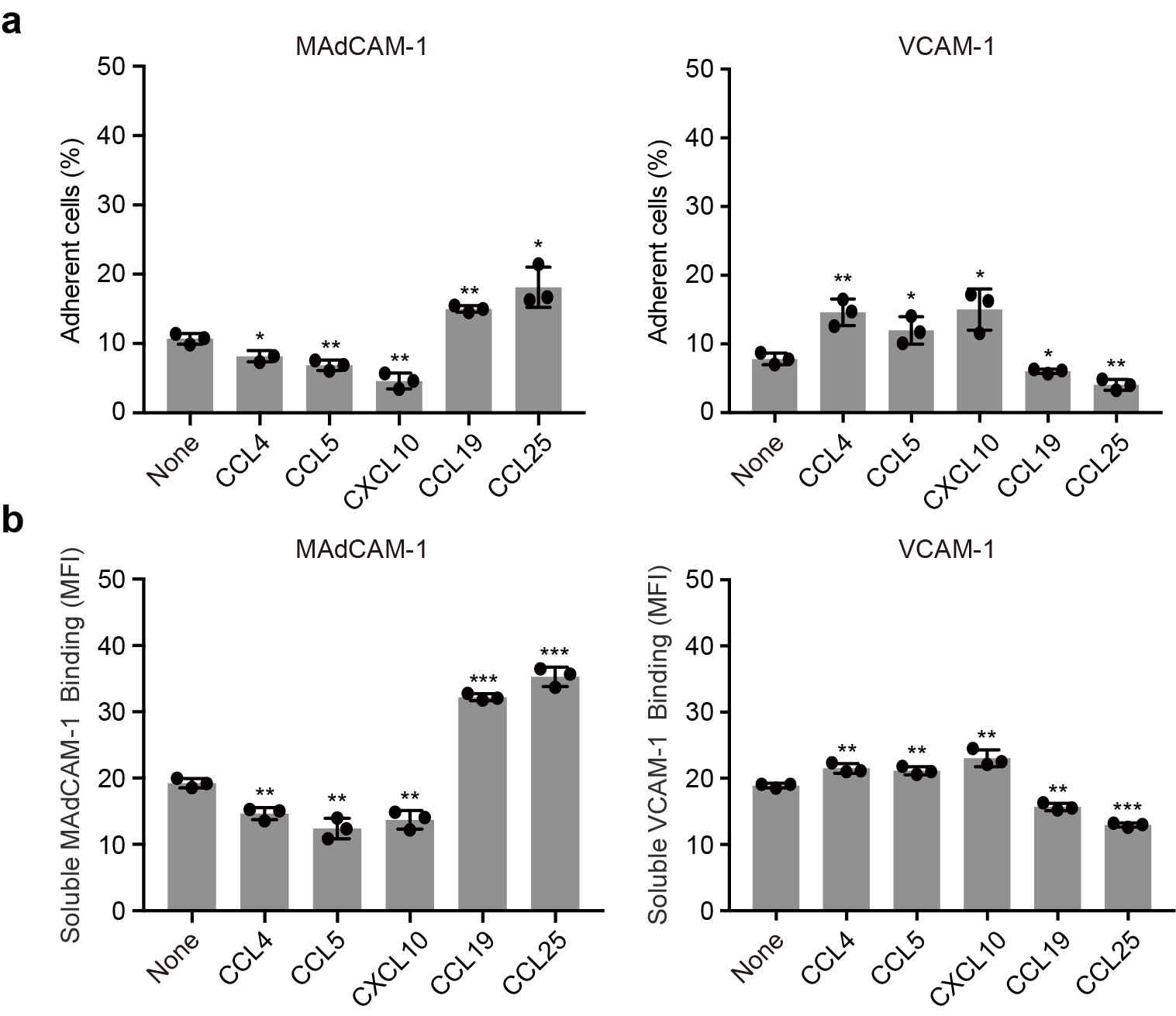


**Fig. S5 Regulation of integrin α4β7-MAdCAM-1 or integrin α4β7-VCAM-1 binding by different chemokines.**

α4β1-VCAM-1 interaction was disrupted by pretreating the cells with β1 blocking antibody AIIB2 (20 μg/ml) for 5 min at 37 °C.

**a** Adhesion of CD4^‒^α4β7^+^-CR Jurkat T cells to the immobilized MAdCAM-1 or VCAM-1 in 1 mM Ca^2+^/Mg^2+^ with and without chemokine stimulation.

**b** Binding of soluble MAdCAM-1 or VCAM-1 to CD4^‒^α4β7^+^-CR Jurkat T cells was calculated with the specific mean fluorescence intensity (MFI) in 1mM Ca^2+^/Mg^2+^ with and without chemokine stimulation.

**Figure S6.**


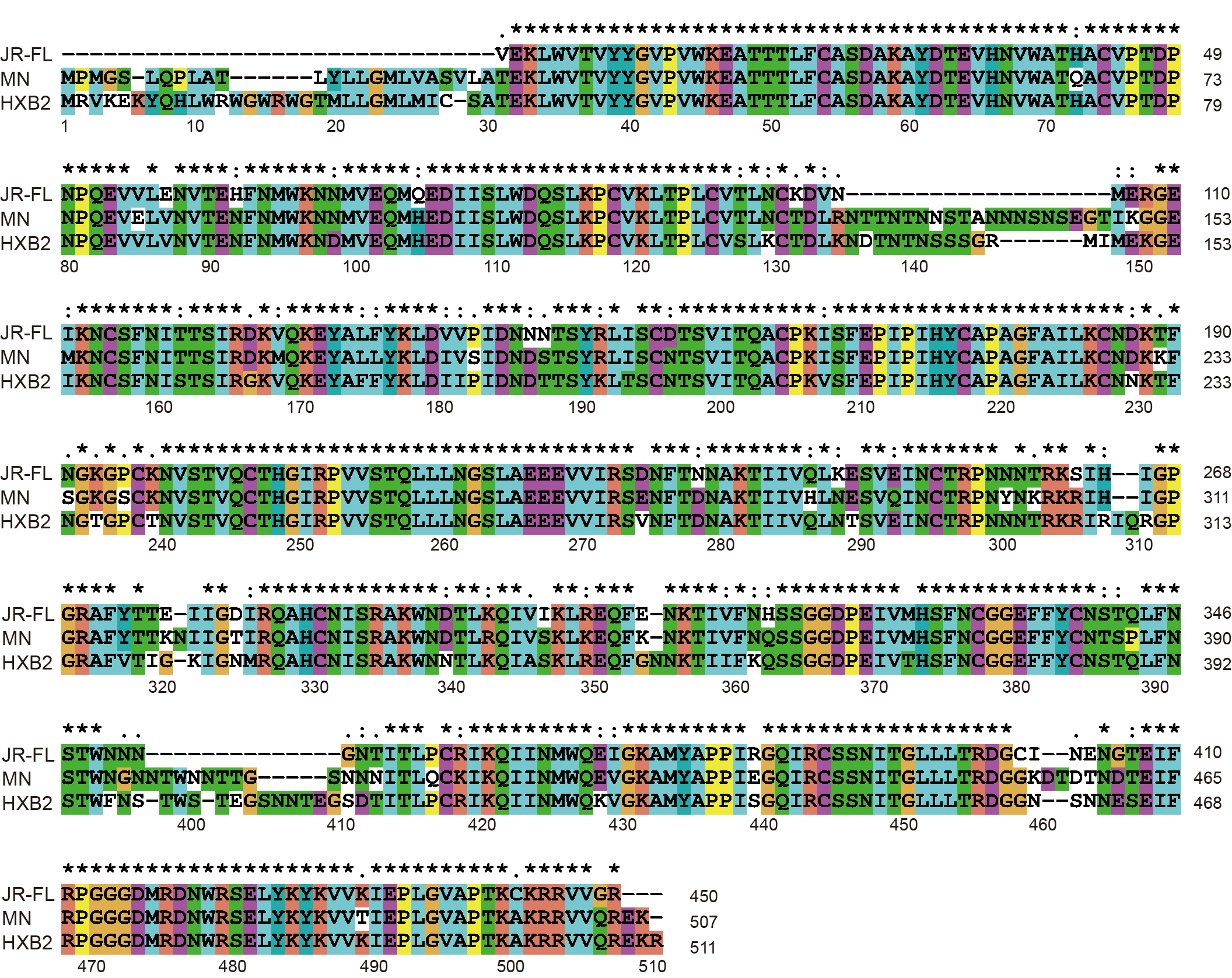


**Fig. S6 Amino acid sequence alignment of three gp120 subtypes.**

The sequence alignment of HIV-1 JR-FL, MN and HXB2. The fully glycosylated HIV-1 envelope glycoprotein trimer JR-FL (PDB: 5FYK) is selected as a template to build the initial conformation of MN gp120 due to the highest 87% sequence identity between JR-FL and MN gp120.

**Figure S7.**


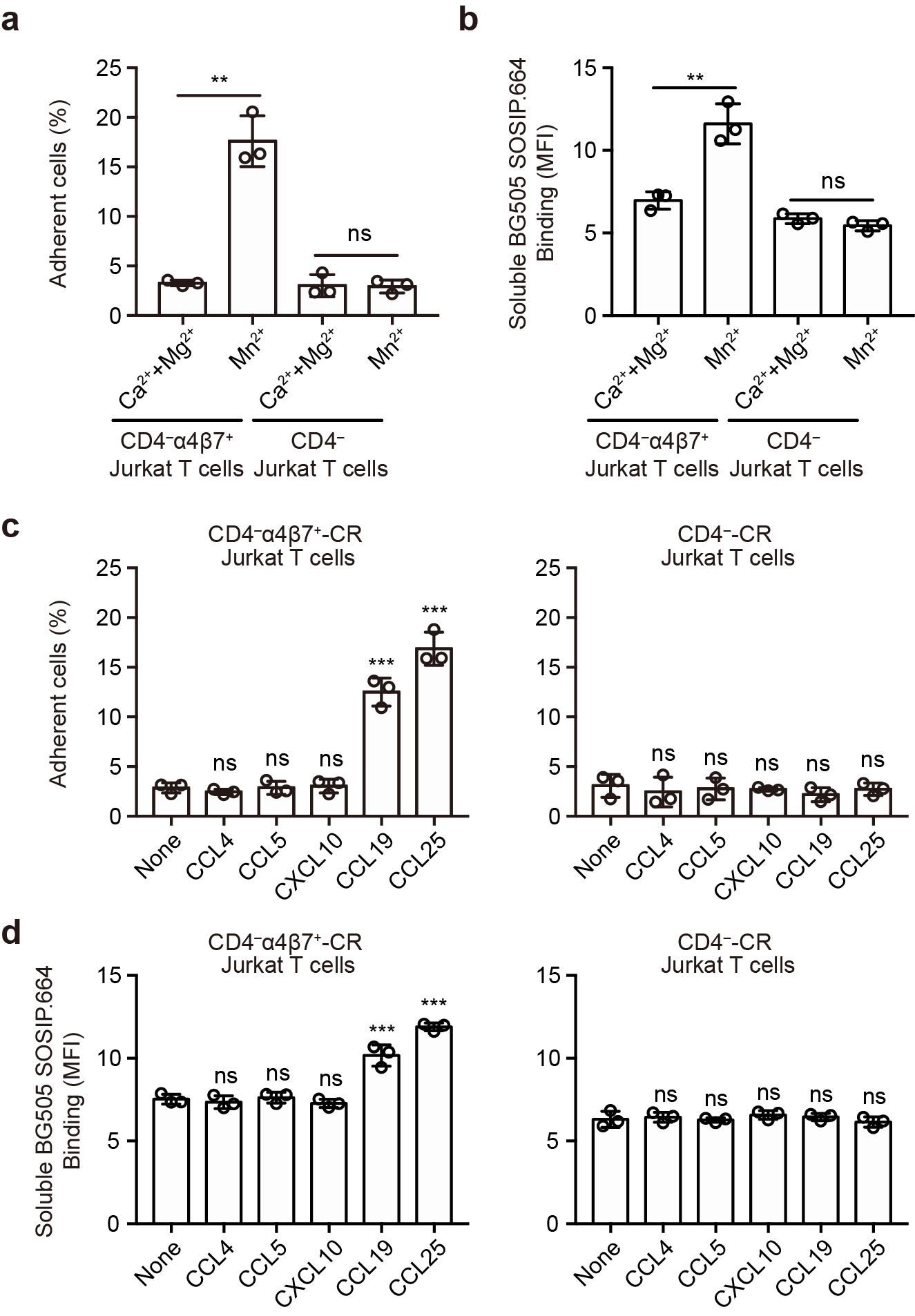


**Fig. S7 BG505 SOSIP.664 binds to the highly activated form of integrin α4β7.**

**a** Adhesion of CD4^‒^ Jurkat T cells or CD4^‒^α4β7^+^ Jurkat T cells to immobilized BG505 SOSIP.664 in 1mM Ca^2+^/Mg^2+^ or 0.5 mM Mn^2+^.

**b** Binding of soluble BG505 SOSIP.664 to CD4^‒^ Jurkat T cells or CD4^‒^α4β7^+^ Jurkat T cells was calculated with the specific mean fluorescence intensity (MFI) in 1mM Ca^2+^/Mg^2+^ or 0.5 mM Mn^2+^.

**c** Adhesion of CD4^‒^-CR Jurkat T cells or CD4^‒^α4β7^+^-CR Jurkat T cells to the immobilized BG505 SOSIP.664 in 1mM Ca^2+^/Mg^2+^ with and without chemokine stimulation.

**d** Binding of soluble BG505 SOSIP.664 to CD4^‒^-CR Jurkat T cells or CD4^‒^α4β7^+^-CR Jurkat T cells was calculated with the specific mean fluorescence intensity (MFI) in 1mM Ca^2+^/Mg^2+^ with and without chemokine stimulation.

**Figure S8.**


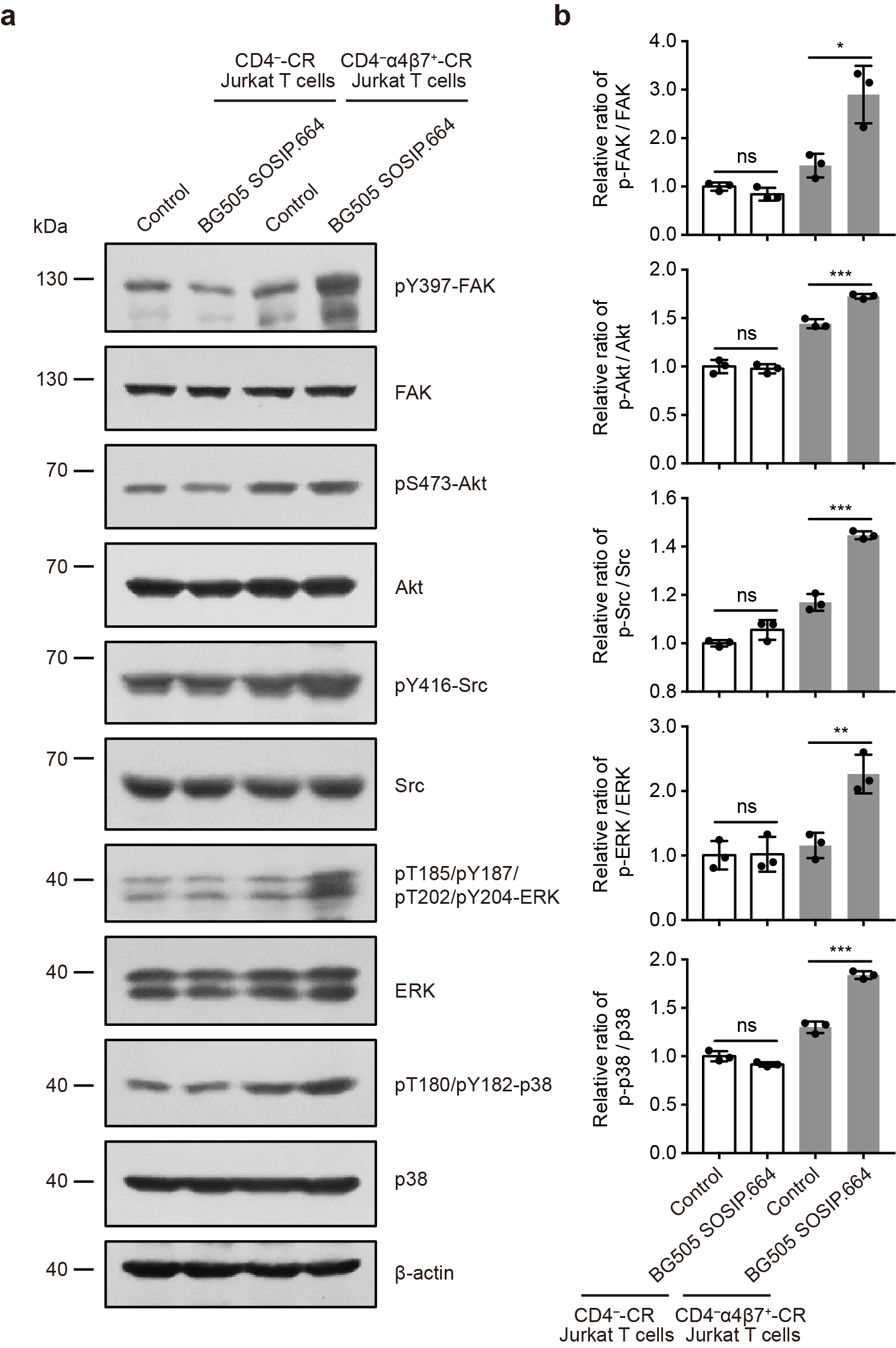


**Fig. S8 BG505 SOSIP.664 activates integrin α4β7 downstream signals.**

CD4^‒^-CR Jurkat T cells or CD4^‒^α4β7^+^-CR Jurkat T cells were pre-treated with CCL25 (0.5 μg/ml, in HBS with 1 mM Ca^2+^/Mg^2+^) for 15 min at room temperature. Then cells were stimulated with BG505 SOSIP.664 (50 μg/ml) for 30 min at 37°C.

**a** The expression and phosphorylation of FAK, Akt, Src, ERK and p38 were determined by immunoblot analysis.

**b** The relative ratios of p-FAK/FAK, p-Akt/Akt, p-Src/Src, p-ERK/ERK and p-p38/p38 were normalized to the values of CD4^‒^-CR Jurkat T cells without stimulation (Control).

**Figure S9.**


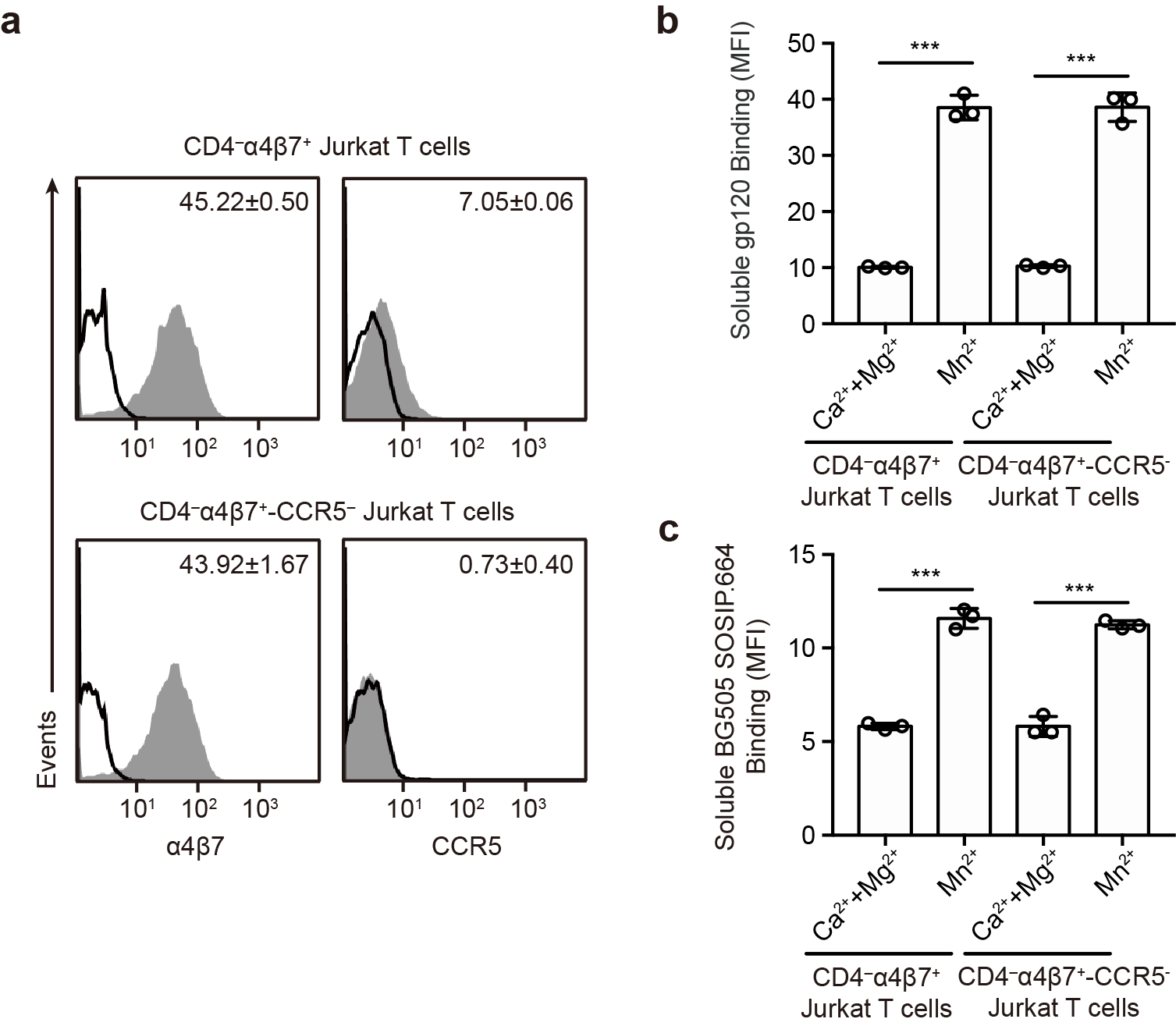


**Fig. S9 α4β7-gp120/BG505 SOSIP.664 interaction is CCR5 independent.**

**a** The expression of α4β7 and CCR5 was determined by flow cytometry. Numbers within the panel showed the specific mean fluorescence intensities. Opened histogram: mock control. CD4^‒^α4β7^+^ Jurkat T cells, upper; CD4^‒^α4β7^+^-CCR5^‒^ Jurkat T cells, bottom.

**b** Binding of soluble gp120 to CD4^‒^α4β7^+^ Jurkat T cells or CD4^‒^α4β7^+^-CCR5^‒^ Jurkat T cells was calculated with the specific mean fluorescence intensity (MFI) in 1 mM Ca^2+^/Mg^2+^ or 0.5 mM Mn^2+^.

**c** Binding of soluble BG505 SOSIP.664 to CD4^‒^α4β7^+^ Jurkat T cells or CD4^‒^α4β7^+^-CCR5^‒^ Jurkat T cells was calculated with the specific mean fluorescence intensity (MFI) in 1mM Ca^2+^/Mg^2+^ or 0.5 mM Mn^2+^.

**Figure S10.**


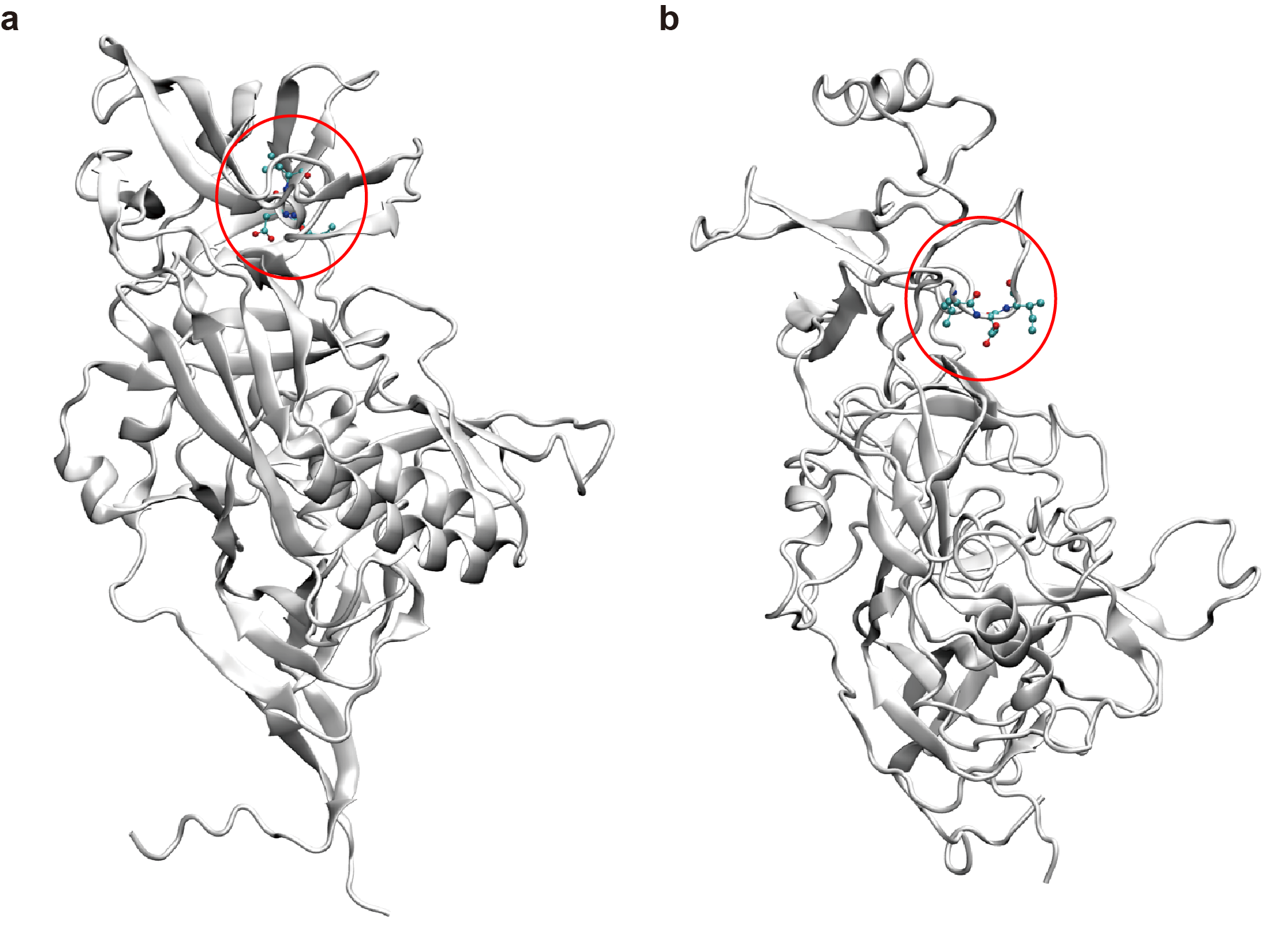


**Fig. S10 Homology model of MN gp120 in the MD simulations.**

The proposed binding epitope L^179^D^180^I^181^ (HXB2 numbering) tripeptide in V2 domain are fully buried in both 5FYK template and MN gp120 mode, which is inaccessible to the metal ion dependent adhesion site (MIDAS) of the β I domain of integrin β7. Hence, Gaussian accelerated MD (GaMD) simulations were conducted to identify the possible solvent-exposed conformation in the L^179^D^180^I^181^ region of MN gp120. After 4 x 800ns GaMD simulations, we observed the L^179^D^180^I^181^ tripeptide in V2 domain of MN gp120 was solvent-accessible. The L^179^D^180^I^181^ tripeptide was indicated in a red circle.

**a** Homology model of MN gp120 before GaMD simulations.

**b** Homology model of MN gp120 after GaMD simulations.
